# Helpful or harmful? Navigating the impact of social media influencers’ health advice: insights from health expert content creators

**DOI:** 10.1186/s12889-024-21095-3

**Published:** 2024-12-18

**Authors:** Jaroslava Kaňková, Alice Binder, Jörg Matthes

**Affiliations:** Department of Communication, University of Vienna, Währinger Strasse 29, Vienna, 1090 Austria

**Keywords:** Social media influencers, Health communication, Health experts, Qualitative interviews

## Abstract

**Background:**

With the growing role of social media influencers (SMIs) in providing health advice, concerns arise regarding the usefulness and reliability of online health information. This exploratory research focuses on health expert content creators (HECCs), who offer a unique perspective due to their combined medical knowledge and social media expertise.

**Methods:**

We conducted semi-structured in-depth qualitative interviews with HECCs to explore their views on SMI-driven health communication, their motivations for participating on social media, and the strategies they employ to counteract misleading health messages on the platforms. The study employed thematic qualitative text analysis to identify key themes and patterns.

**Results:**

HECCs highlighted the complex role of SMIs in public health, acknowledging their potential to promote positive health behaviors while also noting the risks associated with the spread of inaccurate or oversimplified health messages. The findings emphasize the urgent need to broaden health communication research to address not only misinformation but also overgeneralized health messaging, which can be equally detrimental.

**Conclusions:**

The findings underscore the importance of expanding health communication research to address both misinformation and overgeneralized health messaging. Practical recommendations are provided to mitigate the spread of misleading health information by SMIs, informed by the experiences and strategies of HECCs.

**Supplementary Information:**

The online version contains supplementary material available at 10.1186/s12889-024-21095-3.

In the contemporary digital era, the internet, and especially social media, have become crucial sources of health information and advice for an increasingly large segment of the population [1, 2]. Given the projected substantial increase in global social media engagement [3], the role of these platforms in disseminating health information is set to become even more important in the future. Social media influencers (SMIs) are an integral part of the social media landscape and exert a significant influence on the health-related attitudes and behaviors of their followers [4, 5]. Their role in shaping public opinion spans a diverse array of health topics, including but not limited to vaccination, cancer screenings, and fitness [6–8]. The impact of SMIs on public health is mixed; they can significantly contribute to positive health outcomes by promoting vaccination and preventative screenings [6, 7], healthy diet [9, 10], and physical exercise [8, 11]. However, they also have the potential to undermine public health efforts by endorsing unhealthy eating habits [12] and circulating health misinformation [13, 14].

Despite the potential positive impact of SMIs on shaping health-related attitudes and behaviors, alarming evidence suggests widespread dissemination of health-related content by individuals lacking the requisite expertise, often driven by commercial rather than public health interests [4]. This raises significant concerns about the quality of online health information. However, it is crucial to differentiate between non-expert SMIs and health professionals active on social media. Health expert content creators (HECCs) might contribute to desirable health outcomes [15], serving as a bridge between accurate health information and the public. This unique population offers invaluable dual expertise, combining clinical knowledge with firsthand understanding of social media dynamics. Their ability to critically assess the credibility of health content on these platforms provides a nuanced perspective on the spread of potentially misleading health information by their non-expert counterparts. Moreover, their experience in engaging with online audiences, as well as real-life patients, could contribute to understanding potential effective strategies for counteraction.

While qualitative research on health communication of SMIs is limited [4, 16], it seems especially important to explore the specifics and potential effects of SMI-driven health communication in-depth, in order to develop effective strategies to leverage their influence for desirable public health outcomes. Moreover, given the current limited knowledge of HECCs’ attitudes and experiences, a quantitative approach may not sufficiently capture the depth and complexity of their attitudes and motivations, underscoring the necessity of a qualitative approach. To bridge this gap, we conducted semi-structured in-depth interviews with HECCs to explore their experiences and attitudes toward health communication of non-expert SMIs, their motivations for engaging on social media, and their recommended strategies for countering misleading health messages. Additionally, while most research has centered on health misinformation, we advocate broadening future studies to include *overgeneralized health messaging*. This exploratory study confirms the need for this expanded focus, underscore the urgency of addressing this issue, and affirm its comparability to misinformation. By leveraging the unique insights of health professionals who navigate the social media landscape, this study provides a nuanced perspective on the impact of SMIs on public health and offers strategic directions for enhancing the accuracy and impact of digital health information.

## Social media influencers and health

Harff and colleagues [14] describe SMIs as “regular people who became well-known on social media due to their efficient self-presentation on these platforms” (p. 831). In other words, SMIs achieve popularity through their proactive, self-managed presence and personal branding on social media platforms [17]. This factor distinguishes SMIs from traditional celebrities, who usually achieve recognition through industry gatekeepers, such as editors or producers [18]. Nevertheless, the influence of SMIs can surpass traditional celebrities [19]. Additionally, SMIs are often categorized based on the number of followers they have, with classifications ranging from micro-influencers to mega-influencers, depending on the size of their audience. The cut-off points for these classifications can vary across the literature, with different studies applying different follower thresholds [4]. The number of followers SMI has can also influence the effects of their messaging, with micro-influencers, with smaller audiences, being often perceived as more authentic and relatable [20], while SMIs with a high number of followers tend to be seen as more likable and popular [21, 22]. Moreover, SMIs also vary in their focus areas, with some dedicated solely to specific health-related content, such as fitness or nutrition, while others focus on broader lifestyle topics or unrelated issues, yet still occasionally share health-related information [4].

SMIs tend to use a conversational style of communication, which enables them to build close connections with their followers [23], who might perceive them as friends [24, 25]. This phenomenon is attributed to *parasocial relationships* (PSRs) - one-sided relationships where audience members feel a bond with a media persona, despite no direct interaction. Originally described by Horton and Wohl [26], PSRs allow individuals to feel familiar with and emotionally close to a media figure, while experiencing the relationship as evolving [27]. While the influence of SMIs has been extensively studied in the context of advertising and marketing [17, 19, 28, 29], their content is not always driven by marketing objectives but might also often include personal insights and everyday life experiences [30, 31], motivated by SMIs’ desire to build a community and help their followers [32].

The dissemination of health-related information by SMIs is of high significance due to its capacity to shape health perceptions and practices of their audience, thus influencing public views on a broad spectrum of health subjects, such as fitness [8], vaccination [6, 33], or cancer screening [7]. SMIs have been shown to have a positive impact on the exercise intentions of their audience [8, 11], encourage healthy diet [9, 10] or promote positive attitudes toward preventative screenings and vaccinations [7, 33]. However, SMIs can also motivate followers to consume unhealthy food [12] or spread health-related misinformation [13, 14].

### Health experts as social media content creators

Despite concerns regarding the dynamics of patient-physician interactions on social media [34, 35], health experts are increasingly becoming active on these platforms, recognizing the significant potential of their active engagement for public health purposes [36]. In this context, health experts are understood as professionals with advanced knowledge and training in medical or health-related fields, evidenced by education from accredited institutions; examples include medical doctors, nutritionists, or psychotherapists. Health professionals nowadays engage with users on social media to address their personal health concerns, spread knowledge, and assist with lifestyle decisions [37]. Many health experts from a wide array of fields - including neurosurgery [38], plastic surgery [39], or cardiology [40] - have even been recognized as SMIs, due to their success in attracting large followings and exerting substantial influence within their communities. These experts embrace the dual role of being SMIs and health experts in their self-presentation on social media [41], thereby enhancing the public dissemination of health information. Active social media engagement of health experts appears to be beneficial as evidence suggests that they are perceived as more credible and authentic compared to non-expert SMIs [15].

While existing research has explored the motivations behind SMIs’ content creation, ranging from intrinsic motivations like personal enjoyment, to extrinsic motivations such as financial incentives [30], how these motivations translate to other content creators such as health experts, who are increasingly more active on social media, remains underexplored. To address this gap, we ask:

#### RQ1:

What motivates HECCs to create and post health-related content on social media?

Despite the evidence of SMIs’ potential significant influence on the health-related attitudes and behavior of their audience, research suggests that many often disseminate health-related messaging without possessing relevant professional qualifications and give preference to their commercial rather than public health interests [4]. This trend is concerning because as users increasingly turn to social media for health information [1, 2], it raises significant concerns regarding the reliability and accuracy of the health information available there. Given their dual expertise in medical knowledge and social media dynamics, HECCs are uniquely positioned to understand the real-world implications and potential threats posed by misleading health advice disseminated by non-expert SMIs. This leads to our second research question:

#### RQ2:

What are the attitudes of HECCs toward SMIs disseminating health-related information without professional qualification?

### Misleading health information on social media

In recent years, the proliferation of misleading health information on social media platforms has garnered significant scholarly interest [42, 43]. Studies from this field usually use the term health *misinformation*, which can be defined as “information that is contrary to the epistemic consensus of the scientific community regarding a phenomenon” [40, p. 434], or as “a claim that contradicts or distorts common understandings of verifiable facts” [41, p. 10]. It differs from *disinformation* due to the absence of clear intent to deceive its recipients [44].

While the prevalent focus of research is on health misinformation as information that is factually incorrect [44, 45], we argue that this might not be the only form of misleading health messaging potentially leading to adverse outcomes. We base our argument on the fact that human health is an extremely complex concept with no one-size-fits-all solutions, and individual assessment of one’s health condition by a professional is always warranted [46].

Communication of SMIs is a form of mass communication since they typically spread generic messaging to large undifferentiated audiences [47]. However, mass dissemination of identical health messaging to large groups of recipients naturally fails to consider the unique factors influencing individual health-related choices and behaviors of a person [48]. Therefore, we introduce the term *overgeneralized health messaging*, which we define as health-related information that, while factually correct and accurate for the source, possesses the potential to mislead or confuse recipients due to its limited applicability or relevance to a broader audience. This phenomenon can occur when individuals share their personal health experiences, symptoms, or advice, which may lead others to draw inaccurate conclusions about their health conditions or situations.

The concept of overgeneralized health messaging might be exceptionally relevant in the context of health communication of SMIs as these media figures can have a significant impact on their audience’s health-related behaviors [49, 50]. Moreover, on the account of PSR, the generic character of the mass-communicated messaging of a SMI might not be immediately evident to the recipients, who tend to identify with the influencer, viewing them as someone close and similar [17, 29]. As highlighted in broader studies on science communication, generalized messaging can be effective in promoting health-related behavioral intentions across populations despite individual differences [51], which might result in unintended negative consequences in the context of SMI communication.

Research shows that social media influencers (SMIs) routinely share their experiences with both physical and mental health conditions, describing their symptoms, treatment, and personal advice [52, 53]. While this practice can provide valuable insights, it can be also be seen as potentially problematic due to the personal and individual nature of the topic. For example, when SMIs publicly discuss their mental health symptoms, it could lead viewers to self-(mis)diagnose as a result of self-identification with the content creator [54]. Harris and colleagues [55] described that SMIs often generalize their mental health-related personal experiences, which might result in viewers’ self-misdiagnosis and an overreliance on solutions that may not be suitable for everyone. While such messaging is not necessarily incorrect, it can still result in negative consequences like worsening mental health or incorrect treatment [54, 56]. Furthermore, non-expert health advice in general can exacerbate confusion and worsen health conditions [56, 57].

Current research has yet to fully explore the impact of overgeneralized health messaging by SMIs. However, it is crucial first to consult health experts to avoid drawing parallels between two potentially distinct concepts - misinformation and overgeneralized messaging. Therefore, in this study, we aim to examine the outcomes of the two concepts:

#### RQ3:

Are the potentially harmful effects of overgeneralized health messaging by SMIs comparable to those of health misinformation spread by the same individuals?

Furthermore, health experts are on the front lines, directly confronting the consequences of misleading information disseminated by SMIs. Therefore, their experience dealing with the repercussions of such misleading messaging on patient outcomes and public health provides them with unique insights. We argue that this perspective positions them as valuable sources of knowledge on effective strategies to counteract misleading health information. Consequently, this leads to the research question:

#### RQ4:

What measures should be taken to counter the spread of misleading information by SMIs from the perspective of HECCs?

## Method

To investigate the posed research questions, we conducted semi-structured qualitative interviews with experts on both physical and mental health, who are also active social media content creators. The applied method allowed us to gain in-depth understanding of the perceptions, attitudes, and experiences of HECCs regarding potentially misleading health information shared by non-expert SMIs, as well as their recommendations for countering practices and motivations for social media engagement. The research design was approved by the Institutional Review Board of the Faculty of Social Sciences, University of Vienna (approval ID: 20230522_023) and adhered to the ethical guidelines of the American Psychological Association (APA). The research process was reported following the Consolidated criteria for reporting qualitative research (COREQ) (see Supplementary file 1) [58].

### Sample

To find potential participants, we conducted an Instagram search of various profession-related keywords (e.g., “doctor”, “nutritionist”, “psychotherapist”). All terms were searched in both male and female variations to account for the gendered nature of the Czech language. The selection of search terms was based on their relevance to health-related professions and the ongoing identification of key terms throughout the research process, ensuring coverage across various specializations, including both physical and mental health. Based on the results, we compiled a list of potential participants, who were both health professionals and active creators of social media health-related content from Czechia. To qualify as a health professional, individuals had to possess a recognized qualification in a health field, such as a degree in medicine or nutrition, from an accredited institution. For active content creation, individuals needed to have published at least one health-related post on their social media profile every month for the last three months. Suitable candidates were contacted via Instagram messages and, where available, also via email. Altogether, 45 HECCs were contacted. Participants were provided with a consent sheet that included detailed information about the study, including their rights, the voluntary nature of their participation, and how the data would be used. They were asked to review the sheet and provide their signed informed consent prior to participating. The final sample comprised 12 health experts from various fields to enhance the diversity of perspectives in the findings and ensure a broad representation of expertise, including three psychotherapists, three nutritionists, three medical doctors^1^, two pharmacists, and a physiotherapist. Their professional qualification was verified during the interviews. Despite the diversity in their professional backgrounds, saturation was reached with this sample as no new themes or insights emerged from the data. Given the relatively small population size of Czechia, the study’s sample size can be considered proportionate.

Participants were 24 to 42 years old (*M*_*age*_ = 29.67; *SD* = 5.03); nine of them identified as female and three as male. The sample was diverse also in terms of number of followers ranging from 484 to 64,800 *(M =* 14,774.83; *SD =* 20,776.87), thus encompassing HECCs across different scales of social media engagement and further enhancing generalizability. For an overview of the sample, see Table 1.

**Table 1:** Overview of participants

Number	Health Expertise	Number of Instagram Followers ^1^	Gender	Age
#1	Medical doctor (neonatologist)	482	Female	25
#2	Psychotherapist	879	Female	27
#3	Nutritionist	46,200	Male	26
#4	Medical doctor (OB-GYN & sexologist)	64,800	Female	32
#5	Medical doctor (pediatrician)	5,474	Female	28
#6	Nutritionist & medical doctor in training (orthopedist)	2,269	Female	27
#7	Nutritionist	8,127	Female	24
#8	Pharmacist	17,800	Female	42
#9	Psychotherapist	705	Male	30
#10	Pharmacist	5,324	Female	31
#11	Physiotherapist & fitness instructor	1,137	Male	26
#12	Psychotherapist & psychologist	24,100	Female	34

^1^ As of December 2023

### Data collection

The interviews lasted between 26 and 55 min (*M* = 34.83; *SD* = 9.55) and were conducted via Zoom video calls [59]. In one case (#4), the interview was conducted via telephone call per interviewee’s preference. Data collection took place between August 7th and December 18th, 2023. The interviews were conducted by the first author (JK; female), who was a PhD candidate at the time of data collection. JK had received training in both quantitative and qualitative research methods and was the sole member of the research team present during the interviews. Before starting the discussion, JK requested participant consent to digitally record the session. Upon receiving approval, she then obtained oral consent for participation and proceeded with a welcome and personal introduction. JK provided an overview of the study and its objectives, clarifying that it was part of her dissertation research. There was no prior relationship between participants and JK; the only interaction before the interview occurred during the recruitment process.

Following this introduction, JK posed the questions outlined in the interview guide, which was developed by the authors in line with the research questions. The conversations were conducted in Czech, which was the native language of both the participants and the interviewer. The interviews were subsequently transcribed verbatim and translated into English for analysis and reporting. The transcripts were not returned to participants for comments or corrections. The English version of the interview guide is available online: https://osf.io/zbma8/?view_only=36d50595d0be40b88f6c111e6dd83e52.

### Data analysis

The material was analyzed using MAXQDA [60] following the approach to thematic qualitative text analysis as outlined by Kuckartz [61]. This method involves a structured, multi-step process designed to systematically categorize and interpret qualitative data while remaining flexible enough to capture emerging themes. Kuckartz’s approach consists of several stages: familiarization with the data, theme identification, coding, categorization, interpretation, verification, and reporting. This ensures a comprehensive analysis, blending inductive and deductive methods to allow for both predefined research questions and the content of the data itself to inform the analysis.

Initially, JK familiarized herself with the interview transcripts by reading through the material multiple times to immerse herself in the data and identify preliminary overarching themes relevant to the research questions.

Following this initial familiarization, JK proceeded to code the material on a sentence-by-sentence basis, assigning segments of text to relevant categories that emerged from the data. As new overarching themes emerged throughout the analysis, JK revisited the entire dataset to recode the material, ensuring that all relevant data was consistently categorized and aligned with the emerging themes.

Once the coding process was completed, JK interpreted the results by identifying patterns and relationships between the themes. To enhance the reliability and validity of the analysis, JK consulted with co-author (AB) during the verification phase, sharing the transcripts and coding process. AB reviewed the data, provided feedback on the coding, and ensured that the interpretation of the findings was sound and accurately reflected the content.

Finally, the results were synthesized and reported in line with the overarching themes. Participants did not provide additional feedback on the findings after the analysis phase and no repeat interviews were conducted.

## Results

### Motivation for content creation (RQ1)

Our first research question was concerned with HECCs’ motivation for creating and sharing content on social media. Based on the responses of the interviewees, four main motivations were identified, as presented in Table 2.

**Table 2:** Health experts’ motivations for social media content creation

Motivation	Short Description	Sample Quote
*Public Health Education*	Aimed at educating the general public on health matters through social media posts.	“I would also like to educate lay people about medicine because I think that nowadays, the relationship between doctor and patient is not hierarchical as it used to be with the doctor above the patient.” (#5)
*Professional Visibility*	Utilizing social media as a platform for self-promotion to attract potential patients or clients.	“I admit that one of the main motivations was to make myself known and somehow expand my private practice potentially in the future.” (#2)
*Community Engagement*	Focused on creating and engaging with a community; providing a space for sharing experiences, intimate disclosures, and professional feedback.	“The biggest motivation is perhaps a bit selfish, but I really enjoy the interaction with people on social media. I really like when people react to my posts, and I’m a bit sad when I post something, and nobody responds to it. So, I like it when people comment on my content or send me private messages and for example ask questions, or there’s a discussion in the comments section. So that really brings me joy – being able to interact and discuss these things with people.” (#5)
*Misleading Information Counteraction*	Driven by the desire to combat the spread of false health information on social media platforms.	“I got my motivation during the COVID-19 pandemic, during which various misinformation was spread so I thought I could disprove some of it.” (#8)

First, HECCs aim to educate the general public about health-related topics through social media content. The interviewees mentioned focusing on various topics based on their expertise, such as medicine in general (#5), their specific work and qualification (#10), mental health and eating disorders (#2), or addressing the “knowledge gap” (#7) between experts and laypeople regarding nutrition. In the case of mental health, experts emphasized their goal of destigmatizing the topic. One of the interviewed psychotherapists highlighted their approach by stating, “I have chosen the strategy to focus on showing the public that psychotherapy, and generally mental health care, does not have to be a taboo subject and it is a completely normal thing.” (#9).

The second identified motivation highlights HECCs’ recognition of social media as a strategic tool for enhancing public visibility, which, in turn, could potentially expand their client or patient base. This perspective was echoed by most of the interviewees, indicating a widespread acknowledgment of social media’s professional benefits within the health community. For instance, one interviewee articulated the direct advantage by stating, “It is of course also beneficial for me regarding my business because it allows me to reach people who might become my clients because they know me from social media.” (#12). Similarly, another expert noted, “I would be lying if I said it does not have the potential to help me professionally.” (#6).

Furthermore, HECCs acknowledged that their activity on social media is driven by their desire to interact with people and foster a sense of community. Interviewee #2, a psychotherapist specializing in eating disorders, expressed her intention to “build a community of people who maybe can pass on their experiences” (#2), emphasizing the aim to create a supportive space for sharing experiences. Moreover, a gynecologist and sexologist noted that social media acts as a platform for discussing sensitive topics, benefiting from the anonymity and openness of the online community. This professional observed that “people are not shy” and are more willing to discuss matters they might hesitate to bring up in person (#4).

Lastly, HECCs highlighted their motivation to engage actively on social media was to counteract the spread of false health information. For instance, interviewee #3 shared his rationale for starting on social media, stating, “I thought that it would be nice to start fighting with the myths that were actually pouring in on me from all sides on social media and start saying sort of my opinion about it.” (#3).

### Health experts’ attitudes toward health communication of non-expert SMIs (RQ2)

The second research question focused on understanding HECCs’ perceptions of non-expert social media content creators disseminating health-related messaging. The consensus among the interviewees was rather negative, perceiving this trend as both problematic and irritating. One expert mentioned, “I try not to follow many influencers because it makes me really mad when they give health advice” (#8). This sentiment was echoed by others, who stated, “I guess we, as doctors, have a fundamentally united view on this, and that is it bothers us very much, it makes us angry.” (#5), and “I perceive it very negatively.” (#1).

HECCs observed that in many instances, non-expert SMIs tend to offer health-related advice based on their personal experiences while positioning themselves as experts. Women who have experienced childbirth offering advice on women’s health and pediatrics or individuals advising on nutrition following personal weight loss exemplify this trend. One expert highlighted the broader issue of assumed expertise:

“This is kind of an epidemic of inexpertise, where the non-experts act as if they were experts. For example, I am a psychotherapist who really likes coffee. So, I could go and tell people how to make coffee correctly because I am the expert. Well, that is just nonsense, because experience does not equal expertise.” (#9).

Adding to this sentiment, another comment was made: “Everyone who was overweight, then lost some weight and posted about it on social media is now a self-proclaimed expert on nutrition and fitness.” (#1).

Despite the overwhelmingly negative stance toward SMIs providing health-related advice without proper qualifications, some interviewees recognized a positive shift. They noted a trend where SMIs include disclaimers in their posts, stating they are not health professionals and advising their audience to treat their advice accordingly. One expert appreciated this development, saying,

“I feel like there’s a bit of an effort right now when an influencer is talking about mental health, they are talking about their story. That’s where I feel like it’s opening up. I also notice that there’s an effort to add some disclaimer in there that they’re not experts, that it’s totally not their place to pass on some advice, to pass on some support and stuff, which I appreciate.” (#2),

while another added, “I must say I started noticing lately that influencers try to emphasize at least that this is what helped them specifically or was recommended to them by a doctor.” (#8).

### Misleading health-related information shared by SMIs (RQ3)

Despite the prevalence of health misinformation, i.e., factually incorrect information, in discussions about misleading health content on social media, most interviewed HECCs emphasized that information does not necessarily have to be factually incorrect to be misleading and potentially harmful. They underscored the importance of considering the individual needs and conditions of each person. As one expert put it,

“I think that misinformation is not the only type of information that could be harmful to human health. There can also be accurate information that is potentially harmful because it really heavily depends on overall medical condition of the person concerned. It needs to be assessed whether it is applicable to and safe for this specific person.” (#10).

To illustrate the point, interviewees provided numerous examples of what could be described as overgeneralized messaging. This refers to information that, while not factually incorrect, could still be potentially harmful to human health due to its lack of specificity and failure to account for individual health conditions and needs. For instance, a nutritionist expressed her concern over various diets, noting,

“In this case, for example, it really depends on who the person is. For example, if it’s an adolescent, who’s still growing up, I would be very careful with veganism. […] I think that if someone wants to inspire their followers to adopt some alternative diet, such as veganism, they should always also point out all the potential downsides and say that while it might be suitable for one person, it might not be for everyone.” (#7).

Similarly, two interviewees mentioned the popular concept of “what I eat in a day” videos as an example of overgeneralized messaging. These videos, typically shared by SMIs, showcase everything the influencer eats in a day, often as a form of inspiration for their followers. One interviewee explained:

“I think a good example would be the ‘what I eat in a day’ posts. When a nutritionist does that, they always point out that this is based on their own individual needs and goals, and it would look different for other people. So, they bring attention to individual differences, and it serves more as an inspiration. […] However, many profiles do not point these differences out. It’s also common when people share recipes and their portion sizes. Sometimes I see portions and think that for me as an active person, the portion would need to be much larger. I don’t think the majority of the audience realizes this, though. Instead, they may think something is wrong with them if they’re still hungry after eating the meal.” (#7).

Another interviewee added:

“I believe people who don’t know much about nutrition and are desperate to lose weight might take these ‘what I eat in a day’ videos literally - like ‘eat exactly like this if you want to look like me’ - and that can naturally be a big problem.” (#1).

Discussing childbirth experiences shared on social media, an expert pointed out the dangers of oversimplifying complex medical decisions based on personal experience, stating,

“If someone says, ‘I gave birth at home, it was an amazing experience and everything went well,’ that is not misinformation because it might actually be true for them. However, it does not change the fact that home births are extremely dangerous^2^ and if somebody decides to give birth at home based on this good experience of an influencer, this person and their baby do not have to be that lucky and come out of it both healthy and alive.” (#1).

Another expert criticized exercise advice given by SMIs, saying,

“For example, somebody posts a video with ‘the best five exercises for back pain,’ but it is clear that there are no five best exercises for everyone. This influencer says in the video that these are the absolute best five exercises that everyone should be doing, but in reality, while these exercises might be great for some people, they are not going to be suitable and helpful for everyone. It is also very possible that the back pain of some people will get much worse if they do these exercises.” (#11).

Furthermore, all interviewed mental health experts raised concerns about the public disclosure of mental health condition symptoms by SMIs, particularly the risk of self-(mis)diagnosis^3^ this may encourage. Interviewee #2 observed,

“I think that people would self-diagnose based on what influencer says because of their need to belong and identify with someone. So, for example, for adolescents, these young people are trying to find out who they are and where they belong, and if they see that everyone around me who I consider important had some mental illness, then they will want to have one too and probably self-diagnose with one.” (#2).

This sentiment was reinforced by another expert, who remarked, “People try to self-diagnose. For example, if you have this and that, you need to have this diagnosis. Most commonly I feel like people self-diagnose themselves with ADHD for some reason, and usually completely incorrectly.” (#12). The third mental health expert also acknowledged this trend, noting that it is something he encounters also in his practice, and saying that “it is the price for the fact that mental health is now increasingly more discussed.” (#9). Therefore, the public discussion of mental health symptoms can be seen as a form of overgeneralized health messaging, according to the definition used in this study.

Answering RQ3, the interviewees argued that the health-related consequences of misinformation and accurate but inapplicable health advice are generally comparable. One expert elaborated on this point by stating, “I would say that I don’t really see a difference between misinformation and some kind of unsuitable advice in terms of their consequences because in the end, both can lead to very similar results.” (#9). Nevertheless, some experts observed that the two types of messaging may differ in terms of the immediacy with which undesirable consequences manifest. While the adverse health effects of following misinformation can be immediate, the consequences of adhering to overgeneralized advice could emerge over a longer period. One expert explained,

“I’d say the consequences of these two types of information might differ in the speed in which they appear. So, for misinformation it might be quicker and acute, whereas when a person does something like a long-term lifestyle change then the consequences can be damaging to health, but I’d say there’s higher probability somebody will notice and do something about it.” (#7).

To avoid the undesirable consequences of overgeneralized health messaging, HECCs advocated for SMIs to include disclaimers highlighting their lack of professional expertise and the personalized nature of health messaging. They emphasized the importance of recognizing that health conditions vary significantly from person to person. One expert noted, “It’s just extremely important to keep emphasizing that everyone’s journey is different and what worked for one person does not have to work for others.” (#2). Another emphasized the potential risks of overgeneralized advice, suggesting,

“I think it should always be stated there that while these exercises might help some people, they can also be harmful for others so you should always consult a medical professional, who can evaluate their individual case. I think a disclaimer like this would be really helpful because it really can potentially be dangerous and harmful.” (#11).

### Countering measures (RQ4)

Participants provided suggestions that could, from their point of view, help combat the spread of misleading health-related information by SMIs. Overall, five overarching themes were identified, described in Table 3 with corresponding sample quotes.

**Table 3:** Suggested measures to combat spread of misleading health-related information by SMIs

Suggested Measure	Short Description	Sample Quote
*Public Literacy Enhancement*	Focused on improving health and social media literacy among the general public.	“I think the general public should be educated more, so that they do not fall for every nonsense they see online. They should also be constantly reminded about everyone’s individuality when it comes to health. Everyone needs individual assessment and treatment for both their body and mind, and following some general advice is not the way to go.” (#11)
*Education of Content Creators*	Aimed at educating social media content creators to ensure accurate and responsible dissemination of health information.	“I can immediately think of some education for influencers. I don’t know if that’s actually somehow possible, but I think one of the ways to deal with this situation would be to educate influencers on how to talk about mental health and also make them lead them to responsibility for the content they share.” (#2)
*Regulatory Interventions*	Suggesting the implementation or tightening of regulations (legal or social media policy) to curb the spread of misleading health information.	“I think it’s terribly important that the content on social media is monitored - not monitored in the sense that every single sentence is deleted, but a little bit more monitored. A lot of people think that like “this is my profile, yeah 100,000 people follow me, but I can still say whatever I want on here”. So, I think that influencers should be held accountable for what they say on social media so that they think a bit more about what are they putting out and what it can cause. I think the only way to do this is to set stricter rules on social media. Right now, you cannot ever write “sex” on social media, but when people are promoting drinking pool chemicals that’s fine. So, I think this is a huge imbalance.” (#10)
*Authentication of Professionals*	Proposes verifying the credentials of health professionals to establish trust and credibility in their social media content.	“I would really like some kind of verification of professionals so that qualified experts could for example have some badges so that people can see that it is not just anyone posting whatever they want.” (#4)
*Active Engagement of Health Professionals*	Encouraging health professionals to have a more prominent and active role on social media platforms.	“I also really support when health professionals become influencers and they share their expert knowledge on social media, I think this content kind of balances out the misinformation in a way. That actually strikes me as one of the potential solutions, getting more professionals out there. That is something that is already happening from my point of view, and I find it extremely cool.” (#2)

Firstly, there was a strong emphasis on the need to enhance both health and social media literacy to empower individuals to critically assess the health information they encounter online. Alongside this, the importance of educating SMIs on the accurate and responsible dissemination of health information was underscored. Furthermore, participants advocated for the implementation of regulatory interventions. These could take the form of legal measures or policies enforced by social media platforms, designed to limit the spread of misleading health content. In addition, the value of health professionals maintaining an active presence on social media was recognized as a key factor in improving the situation. By directly engaging with the public, experts can offer accurate information and effectively counteract misleading information. In relation to this, health experts suggested an official social media verification system based on qualifications. Such a mechanism would help users easily identify genuine health experts, thereby enhancing the credibility of the health information shared and making it easier for the public to distinguish between qualified and unqualified sources.

## Discussion

This study aimed to provide in-depth insights into the HECCs’ perspectives on non-expert SMIs disseminating health information, especially concerning potentially harmful and misleading messaging. First, we identified four primary motivators for health experts’ active engagement with social media: (a) they are driven by a commitment to public health education, aiming to share accurate health information and advice with the broader public; (b) they are interested in building and engaging with communities on social media, fostering spaces for dialogue and support around health topics; (c) they proactively combat the spread of misleading health information; and (d) they recognize the potential personal benefits of public visibility and effective self-promotion, acknowledging that an active social media presence can enhance their professional reputation and opportunities. These motivations help explain the growing numbers of health experts turning to social media to extend their influence beyond traditional medical settings, as evidenced by recent research [39, 67]. Since social media users search for health-related information on these platforms [68], it is desirable to increase the presence and active engagement of health experts on social media [69]. By understanding the motivators for health experts’ participation on social media, agencies can develop targeted strategies and support systems to facilitate and enhance the online activity of health professionals.

Next, our study revealed a general skepticism among HECCs toward non-expert SMIs. This skepticism arises from concerns that personal health-related experience, frequently shared by non-expert SMIs, might often be mistakenly equated with expertise, potentially leading to the spread of misleading information and harmful advice. These apprehensions resonate with the broader discourse on the destabilization of traditional expertise in the digital era, where lived experiences and peer-to-peer advice on social media platforms are increasingly valued over formal qualifications and medical training [70–72]. These findings align with concerns highlighted by previous studies, which noted that SMIs often share health advice on social media without the necessary expertise, and might prioritize commercial interests (e.g., promoting unhealthy food products) over public health concerns [4]. While the observation that some non-expert SMIs increasingly include disclaimers represents a positive shift - indicating a growing awareness of the limitations of personal experience in offering health advice and the potential impact of SMIs’ influence on their followers’ decision-making and lifestyle choices - research in the context of back pain-related TikTok content found that creators sparingly encouraged viewers to seek professional help [73]. In this context, collaborations between SMIs and health professionals, such as the cooperation of British SMI Zoe Sugg with Jo’s Cervical Cancer Trust^4^, could present a valuable opportunity to contribute to the spread of accurate and beneficial health information [74], and should be explored by further research.

Furthermore, in this study, we introduced the term *overgeneralized health messaging* to describe information that while technically accurate, could be potentially misleading and result in undesirable health outcomes, paralleling the effects of health misinformation. HECCs emphasized that health information does not need to be factually incorrect to be detrimental, especially when disseminated by SMIs, with whom audiences often develop close, friend-like relationships [24, 25]. This underscores the importance of context and individual health needs in evaluating the accuracy and helpfulness of health information, challenging the binary of true/false in assessing the value and impact of health content on social media.

As highlighted by the HECCs, this potentially harmful messaging can manifest in various ways, such as sharing personal dietary or exercise routines, or discussing individual experiences with health conditions by detailing symptoms and treatments. The latter seemed to be particularly relevant to mental health, where all interviewed mental health experts expressed concern over the potential for such disclosures to lead to self-(mis)diagnosis among followers. This aligns with previous news reports [54, 56] that public discussions of mental health symptoms could lead to confusion, self-(mis)diagnosis, and inappropriate treatment choices. Nevertheless, this concern also extends beyond mental health to physical health, as previously explored by journalists as well [57].

In contrast to SMI messaging, overgeneralized health messaging also appears in other contexts, such as peer support groups. However, in these communities, the advice is typically solicited, and members share actual similarities, seeking experiential advice from individuals with similar conditions [75]. Sillence [75] further highlights, that advice in peer support groups often includes recommendations to seek professional medical guidance, with members specifically requesting advice from individuals whose health circumstances closely mirror their own. Therefore, while advice in peer support groups may still suffer from inaccuracies, especially when it lacks professional medical guidance, it is likely more relevant to an individual’s specific health situation. Conversely, the perceived closeness and similarity with SMIs is often illusory, based on PSRs [17, 29]. The risk here parallels findings from broader research on politicized scientific communication, where generalized messages can have widespread effects across diverse audiences due to their broad, untargeted nature, which may however overlook individual needs and context [51]. As a result, overgeneralized health messaging from SMIs may pose a greater risk of harm due to its broader reach, lack of tailored relevance, and potential to mislead audiences. Moreover, the interviews highlight that overgeneralized messaging by SMIs can manifest in various forms, not limited to direct health advice, such as symptom disclosures, showcasing personal exercise or dietary regimens, or sharing personal experiences with health conditions. These different types of messaging warrant further exploration in future research. Our study is the first empirical work to describe the phenomenon of overgeneralized health messaging by SMIs and validate its significance through the perspectives of HECCs.

Moreover, this study revealed that, according to the HECCs, the adverse consequences of health misinformation and overgeneralized health messaging can be considered essentially comparable in severity, despite the latter being factually accurate. Nevertheless, it was observed that in some cases, the repercussions of overgeneralized health messaging might not manifest as immediately as those resulting from misinformation. The conceptualization of overgeneralized health messaging highlights a critical pathway for future research to probe into the intricacies of overgeneralized health messaging disseminated by SMIs. Such inquiries should aim to delve deeper into the specific characteristics, consequences, and comparability of overgeneralized health messaging to health misinformation. We argue this exploration is vital for developing effective communication strategies and interventions to counteract the nuanced challenges posed by the dissemination of misleading health information on social media.

The consensus among HECCs emphasized the critical need for educating both audiences and content creators, augmented by regulatory measures to ensure the accuracy of health information shared online, echoing recommendations from research [4]. In addition, the active engagement of health experts on social media platforms was proposed as a key strategy for mitigating the spread of misleading health information. Their visible presence is not only desirable but also potentially effective in counteracting misleading content, given that health professionals are regarded as more credible and authentic sources of information compared to non-expert SMIs [15, 69]. Moreover, the suggestion to implement a verification process for health experts’ social media accounts presents a practical tool to enhance the visibility and influence of credible health information sources. By verifying professional credentials and marking their profiles accordingly, social media platforms could significantly aid in distinguishing qualified health experts from non-experts. In line with this, platforms like YouTube have introduced initiatives such as YouTube Health^5^, which fosters cooperation between the platform and health professionals and increases the visibility of authoritative health content by featuring verified health professionals and institutions. While the effectiveness of such interventions in the context of health experts on social media is yet to be examined, existing research presents mixed findings. On one hand, Edgerly & Vraga [76] found that the credibility of a user is not significantly influenced by the presence of a verification badge. On the other hand, recent studies, such as that by Liao et al. [77], suggest that verification badges enhance trust in posts. Therefore, we encourage future research to delve into this issue as understanding the nuanced effects of verification on the perceived credibility and trustworthiness of health experts’ social media accounts is crucial for developing effective strategies to combat misleading health information spread on the platforms.

### Limitations

First, the interviews were conducted exclusively online or via phone calls, precluding the ability to observe non-verbal cues such as body language, which could offer additional insights into participants’ responses. Second, the research focused solely on health experts active on Instagram, potentially overlooking the perspectives of health professionals who utilize other platforms, such as TikTok [78], which might exhibit different dynamics and audience engagements. Next, the country of data collection needs to be considered when interpreting the results. Specifically, the interviews were conducted in Czechia, an EU member state with social health insurance system with universal membership, ensuring widely accessible health services at no direct cost to citizens [79]. The country’s healthcare system could potentially influence both the health information seeking behaviors on social media and the motivations behind HECC’s social media engagement.

Additionally, the study likely encountered self-selection bias, as potentially only those experts who had an existing interest in the topic agreed to participate, possibly skewing the sample. Finally, while most of the HECCs described their social media engagement as an altruistic effort, the possible social desirability bias needs to be taken into account when interpreting the results.

## Conclusion

This exploratory study identified primary motivators for health experts’ active engagement with social media and revealed a general skepticism among HECCs toward non-expert SMIs and their health-related communication. There exists a critical need to broaden the scope of health communication research to include overgeneralized health messaging, which can be just as detrimental as outright misinformation. HECCs recognized the importance of context and individual health needs in evaluating health information and validated concerns over the potential for overgeneralized messaging to lead to self-(mis)diagnosis and inappropriate health decisions, emphasizing its comparability in severity of its consequences to misinformation. They highlighted the necessity of educating both audiences and content creators, alongside regulatory measures, and verification of health experts’ social media accounts to ensure the accuracy of online health information.

## Electronic Supplementary Material

Below is the link to the electronic supplementary material.

Supplementary Material 1

## Data Availability

The data collected and analysed during the current study are available from the corresponding author upon reasonable request.

## Footnotes

1 One of the participants was a certified nutritionist as well as a medical doctor in training.

2 This quote represents personal opinions and experiences of the interviewee. While home births are associated with certain risks, they also offer health-related benefits and can be considered suitable for low-risk pregnancies when attended by qualified professionals. Home births remain a controversial topic for many health professionals, with perspectives varying depending on factors such as access to healthcare and the specific circumstances of the pregnancy [62, 63].

3 While the potential for self-(mis)diagnosis of mental health conditions following exposure to overgeneralized messaging by SMIs was mentioned multiple times by interviewees, based on their own experiences and opinions, research in this area remains inconclusive. More evidence is needed to fully understand the relationship between social media and mental health outcomes [64–66].

4 https://www.youtube.com/watch?v=2uceXJ3d6xY.

5 https://health.youtube.

## Abbreviations

HECC: Health expert content creator
SMI: Social media influencer
PSR: Parasocial relationship
e.g.: Exempli gratia, for example
i.e.: Id est, that is
et al.: Et alii, and others
M: Mean
SD: Standard deviation
JK: Jaroslava Kankova (first author)
AB: Alice Binder (second author)
